# A randomised phase II trial to examine feasibility of standardised, early palliative (STEP) care for patients with advanced cancer and their families [ACTRN12617000534381]: a research protocol

**DOI:** 10.1186/s40814-019-0424-7

**Published:** 2019-03-14

**Authors:** Jennifer Philip, Anna Collins, Brian Le, Vijaya Sundararajan, Caroline Brand, Susan Hanson, Jon Emery, Peter Hudson, Linda Mileshkin, Soula Ganiatsas

**Affiliations:** 1 Department of Medicine, University of Melbourne, c/o St Vincent’s Hospital, Victoria Pde, Fitzroy, 3065 Australia; 2 Palliative Care Service, St Vincent’s Hospital Melbourne, Fitzroy, Australia; 3 Palliative Care Service, Royal Melbourne Hospital, Parkville, Australia; 4 Palliative Care Service, Peter MacCallum Cancer Centre, Melbourne, Australia; 5 Public Health, La Trobe University, Bundoora, Australia; 6 Department of Epidemiology and Preventive Medicine, Monash University, Melbourne, Australia; 7 Cancer Australia, Surry Hills, Australia; 8 Department of General Practice, University of Melbourne, Melbourne, Australia; 9 Centre for Palliative Care, St Vincent’s Hospital Melbourne, Melbourne, Australia; 10 Department of Medical Oncology, Peter MacCallum Cancer Centre, Melbourne, Australia

**Keywords:** Palliative care, Integrated care, Family caregivers, Intervention, Trial, Quality of life, Unmet need

## Abstract

**Background:**

Current international consensus is that ‘early’ referral to palliative care services improves cancer patient and family carer outcomes; however, in practice, these referrals are not routine. Uncertainty about the ‘best time’ to refer has been highlighted as contributing to care variation. Previous work has identified clear disease-specific transition points in the cancer illness which heralded subsequent poor prognosis (less than 6 months) and which, we contest, represent times when palliative care should be routinely introduced as a standardised approach, if not already in place, to maximise patient and carer benefit. This protocol details a trial that will test the feasibility of a novel standardised outpatient model of early palliative care [Standardised Early Palliative Care (STEP Care)] for advanced cancer patients and their family carers, with referrals occurring at the defined disease-specific evidence-based transition points.

The aims of this study are to (1) determine the feasibility of conducting a definitive phase 3 randomised trial, which evaluates effectiveness of STEP Care (compared to usual best practice cancer care) for patients with advanced breast or prostate cancer or high grade glioma; (2) examine preliminary efficacy of STEP Care on patient/family caregiver outcomes, including quality of life, mood, symptoms, illness understanding and overall survival; (3) document the impact of STEP Care on quality of end-of-life care; and (4) evaluate the timing of palliative care introduction according to patients, families and health care professionals.

**Methods:**

Phase 2, multicenter, open-label, parallel-arm, randomised controlled trial (RCT) of STEP Care plus standard best practice cancer care versus standard best practice cancer care alone.

**Discussion:**

The research will test the feasibility of standardised palliative care introduction based on illness transitions and provide guidance on subsequent development of phase 3 studies of integration. This will directly address the current uncertainty about palliative care timing.

**Trial registration:**

Australian New Zealand Clinical Trials Registry ACTRN12617000534381.

## Background

Patients with advanced cancer suffer numerous distressing physical symptoms, psychological morbidity and unmet information and psychosocial needs [1–4]. These patients assign high priority to symptom relief, alongside collaborative decision making and open communication [5]. Despite this, gaps remain in translating these preferences into routine care. Patients reporting pain (51.8%) and breathlessness (29.7%) on symptom screening tools prompted health professional responses in just 16.9% and 3.9% of cases, respectively [6]. In practice, symptom screening itself occurs inconsistently, [7] suggesting discrepancies between symptom prevalence and professionals’ responses may be even greater [8]. Family carers of advanced cancer patients also report needs for information, practical and psychosocial support, which are inconsistently addressed [9, 10]. New models of care delivered in a standardised fashion are required to meet the needs of advanced cancer patients and their family carers, and decrease care variability.

Palliative care aims to improve quality-of-life through attention to symptoms, psychosocial needs, information provision and family support. Benefits of timely referral to palliative care services for advanced cancer patients include improved symptom management, quality-of-life and care satisfaction; reduced rates of hospitalisation and emergency department presentations [11–17]; and improved quality-of-life and care satisfaction for family carers [9, 10, 18, 19].

With the ageing of the population, the incidence of cancer is increasing alongside the demand for palliative care [20]. Efficient, equitable and sustainable models of palliative care are required. Outpatient palliative care clinics represent such a model enabling consultation with greater numbers of patients (compared to community based and inpatient services); enhanced capacity for timely response; and hence improved equity of access to palliative care. Outpatient models of care are also consistent with the delivery of continued anticancer treatments [21].

The current international consensus is that ‘early’ referral to palliative care services improves cancer patient and family carer outcomes [17, 22, 23]; however, in practice, these referrals are not routine [24]. Our earlier work demonstrated only 59% of Victoria, Australia decedents with metastatic non-small cell lung, small cell lung, prostate and breast cancers received a palliative approach to care, and for 61%, referral only occurred in the final hospital admission concluding in death [24]. Therefore, it appears that despite a diagnosis of poor prognostic disease, there is no routinely timely access to palliative care. This means that physical, emotional and psychosocial needs may remain unaddressed, and the expert communication, which facilitates patients’ planning and spending their final phase of life in a manner of their choosing, may therefore also be unavailable.

A series of barriers to palliative care referral have been identified, including concerns about difficulty of referral, fear of destroying patient hope associated with perceptions of palliative care [25] and uncertainty over the ‘best time’ to refer [26]. A standardised evidence-based model of early palliative care referral will minimise barriers by providing reassurance about the quality of care, clarifying the times at which referrals are indicated and, increasing patient acceptance of referrals, given it represents ‘routine’ care [27].

The magnitude of benefits around early palliative care referrals varies between studies and is explained by shortcomings in study design, sample size and analyses, as well as differences in definitions of early referral [17]. Studies have (1) variably followed recommended guidelines for the development and testing of complex interventions, [17, 28–31] (2) and no randomised trial has, to our knowledge, investigated disease-specific evidence-based transition points serving as the prompt for palliative care referral or the (3) full economic cost implications of such models. Definitions of early palliative care referral may differ between tumour groups and, to date, a ‘one-size-fits all’ approach has been employed. Yet, the development of bone metastases in the patient with lung cancer, for example, confers a substantially different prognosis compared to the same sites of metastases in a patient with breast cancer. There is a clear need for the testing of cancer-specific time points when referral to palliative care occurs as ‘standard quality care’ [24].

We recognise the cogent position that states palliative care should be introduced based upon the level of need, and acknowledge the important work that has occurred to develop needs assessment instruments which may be used to determine who to refer to palliative care [32]. Yet, uptake and application of these instruments is limited, with clinicians not routinely screening for needs, and consequently many patients are referred late or not at all [8, 33].

Studies by the investigators using coded hospital discharge datasets have examined health care use for patients with high grade glioma (HGG) and metastatic breast, prostate and lung cancers [15, 24, 34, 35]. This work demonstrated clear disease-specific transition points in the illness trajectories which heralded subsequent poor prognosis (less than 6 months and subsequent increased health service utilisation) [24]. These transition points represent times when we predict that palliative care should be routinely introduced, if not already in place, to maximise patient and carer benefit. We sought to use triggers for palliative care that do not rely upon individual clinician engagement, but instead are linked with administrative systems of health service provision and may therefore occur in a routine, equitable way to augment clinician-based decision-making.

We present the protocol of our trial that will test the feasibility of a novel standardised outpatient model of early palliative care [Standardised Early Palliative Care (STEP Care)] for advanced cancer patients and their family carers, with referrals occurring at the defined disease-specific evidence-based transition points. Testing integration of palliative care based upon transition points makes this a world-first study, with resulting data used to refine the model and data collection processes, identify issues with participant consent/retention, calculate effect sizes to inform a phase 3 trial to establish patient/carer benefits and cost implications and evaluate the timing of palliative care introduction according to patient and family carers.

## Methods

### Primary aim

1. To determine the feasibility of conducting a definitive phase 3 randomised trial, which evaluates effectiveness of STEP Care (compared to usual best practice cancer care) for patients with advanced breast or prostate cancer or HGG. The specific feasibility endpoint is defined as enrolment of 90 patients across three sites in 24 months, with at least 60% of patients progressing to study completion, defined as 12 weeks post baseline. This will inform the planning of a subsequent phase 3 study including numbers of centres necessary to recruit the required sample size.

### Secondary aims

1. To examine preliminary efficacy of STEP Care on patient outcomes, including quality of life, mood, symptoms, illness understanding and overall survival.
2. To examine preliminary efficacy of STEP Care on caregiver outcomes, including quality of life, mood, preparedness to care and satisfaction with care.
3. To document the impact of STEP Care on health service use in the last month of life based on parameters of established quality end-of-life indicators, including hospital and intensive care length of stay, emergency department admissions, chemotherapy use and place of death.
4. To evaluate the timing of palliative care introduction according to patients, families and health care professionals.

The key secondary outcome of effect upon quality of life will enable the appropriate sample size calculation for a robust phase 3 trial.

### Design

This study will involve a phase 2, multicenter, open-label, parallel-arm, randomised controlled trial (RCT) of STEP Care plus standard best practice cancer care versus standard best practice cancer care alone. This RCT has been designed within the Medical Research Council (MRC) framework for the development and testing of complex interventions [28, 31]. The MRC framework prioritises phased, sequential and intervention development leading towards implementation [28, 31]. Thus, this study is underpinned by the investigators’ earlier exploratory data resulting from qualitative [9, 26, 36, 37] and phase 1 modelling studies [15, 24, 34, 38] to define transition points where palliative care should, at minimum, be integrated. The transition points, defined by our previous work which will be used as the time of integration in this RCT, are presented in Table 1. Of note, these transition times are viewed as a minimum standard of care, with palliative care integration implemented if it has not already occurred. Consistent with the MRC framework, this phase 2 trial tests the feasibility of implementing palliative care at these transition points, before proceeding to a definitive phase 3 trial.

**Table 1:** Transition point definitions

Prostate cancer	Presence of metastatic disease and multi-day hospital admission
Breast cancer	Presence of visceral metastatic disease (metastases involving organs other than bone only) and multi-day hospital admission
High grade glioma	Hospital presentation (inpatient or outpatient): first recurrence of primary HGG where pathological or clinical diagnosis is glioblastoma/WHO grade IV disease or first diagnosis of primary HGG and no cancer specific treatment being prescribed

### Study setting

This study is being undertaken at three metropolitan tertiary cancer services in Melbourne, Victoria, the second largest state of Australia. These hospitals have specialist palliative care providing both inpatient and outpatient consultation services. A Consumer Advisory Committee was established for the duration of the trial to work simultaneously with the chair of the project research team, ensuring relevance and dissemination of the trial outcomes to included patient groups.

### Funding

Funding for this trial was obtained from the Victorian Cancer Agency via a competitive health services research grant [HSR15022].

### Ethics and safety reporting

Central ethical approval for the trial conduct at all participating sites was provided by the Human Research Ethics Committee at St Vincent’s Hospital Melbourne [HREC 179/16]. The trial was registered with the Australian and New Zealand Clinical Trial Registry [ACTRN12617000534381]. Safety reporting procedures were established and recorded according to protocol.

### Participants

#### Inclusion criteria for patients

Patients included were adults with advanced prostate or breast cancer or HGG who attend a participating hospital at time of defined cancer-specific transition points (Table 1), defined as admission for a multi-day hospitalisation and any metastases (prostate cancer), visceral metastases (breast cancer), recurrence/progression of HGG or diagnosis of HGG when no cancer specific treatment is being prescribed. All patients must be able to provide informed consent and comply with study procedures. Exclusion criteria for patients include those less than 18 or previously seen by hospital consultancy palliative care services within the previous 12 months, or those identified more than 30 days following the identified cancer-specific transition point as described above.

#### Inclusion criteria for family carers

Family carers were eligible if nominated by the patient as their primary support person and able to provide informed consent and comply with study procedures. Exclusion criteria for family carers included being aged under 18 or not willing to be considered the primary family carer.

### Study procedures

#### Recruitment and consent

Consecutive eligible inpatients and outpatients from three Victorian hospitals will be approached for potential study inclusion by research staff. Eligibility data will be recorded along with reasons for refusal to participate. Patient recruitment will occur as soon as possible after the patient has been identified as reaching a transition point (as defined). The research nurse will screen relevant inpatient admission and outpatient lists for potentially eligible patients. Clinical teams will then confirm eligibility and seek permission from the patient to provide information about the study. Provision of a Plain Language Statement and Consent form will be made. Those patients willing to proceed will sign a consent form for the study including consent for access to health service use data. Patients will also be asked to identify a family carer and provide consent for the research nurse to contact them about the study.

Nominated carers will be contacted by the research nurse and invited to participate in the study. The research nurse will provide and explain in detail the carer with a copy of the Plain Language Statement and Consent form either in person or by phone if contact has been made using that mechanism. In the latter case, the hard copy consent form will be sent and returned (signed) by post.

#### Randomisation

Patient-level randomisation will be centralised and coordinated by the trial coordinator who, in real time, accesses the independent blinded system, separate to the treating clinical and patient-interfacing research staff. The randomisation schedule involved 1:1 allocation and used the minimisation method to ensure a balanced distribution between groups with respect to the patient’s tumour type and hospital site. After consent and baseline data is obtained, the research nurse telephones the trial coordinator for the outcome of the randomisation process. The research nurse will liaise with the treating clinician to inform them of the outcome of randomisation.

#### Usual care: standard best practice cancer care

All patients will receive usual oncological care through their usual health care providers. This may include systemic therapy, radiotherapy, surgery or other treatments deemed appropriate. In addition, those patients randomised to usual care may be referred for usual palliative care services at any time deemed appropriate at the treating clinician’s discretion. The timing of any palliative care utilisation will be recorded as part of monthly data collection processes.

#### Intervention: STEP Care plus standard best practice cancer care

Those patients randomised to the intervention arm will receive STEP Care in addition to standard best practice cancer care. STEP Care consists of, at minimum, monthly palliative care consultations and follow up for at least 3 months (in total, minimum of four consultations—initial consultation plus follow up for 3 months). These consultations will be primarily based in the outpatient setting, unless coinciding with a hospital admission, in which case the consultation can be conducted as an inpatient.

All STEP Care consultations will be conducted by an accredited palliative care physician or specialist nurse. At each consultation, a series of domains will be reviewed and activities undertaken (Table 2). Those areas covered in each consultation will be recorded in a standardised manner using the framework provided by the PC-NAT-PD [39]. All STEP consultations will be audio-recorded to enable content review of services delivered. An audit of 20% of all audio-recorded consultations and associated documentation using the Needs Assessment Tool: Progressive Disease–Cancer (NAT: PD-C) [39] will be undertaken based on consecutive cases.

**Table 2:** Key components of STEP Care intervention

1. Identification of patients for eligibility at standardised transitions in the illness course	
2. Initial hospital based palliative care consultation, addressing:	
(a) Review of underlying disease management	
(b) Screening for symptom distress	
(c) Screening for psychological distress	
(d) Review of informal social supports	
(e) Review of formal community supports, including local community palliative care	
(f) Providing information	
(g) Advance care planning discussions	
(h) Involvement of family carer, including enquiry of concerns, needs for information	
3. Regular follow-up, at minimum monthly for minimum of 3 months	
4. Case conference with the general practitioner within 28 days, addressing	
(a) Current and anticipated problems	
(b) Recommended management and therapies	
(c) Designation of responsibility for different aspects of care	

#### Data collection

**Fig. 1:**
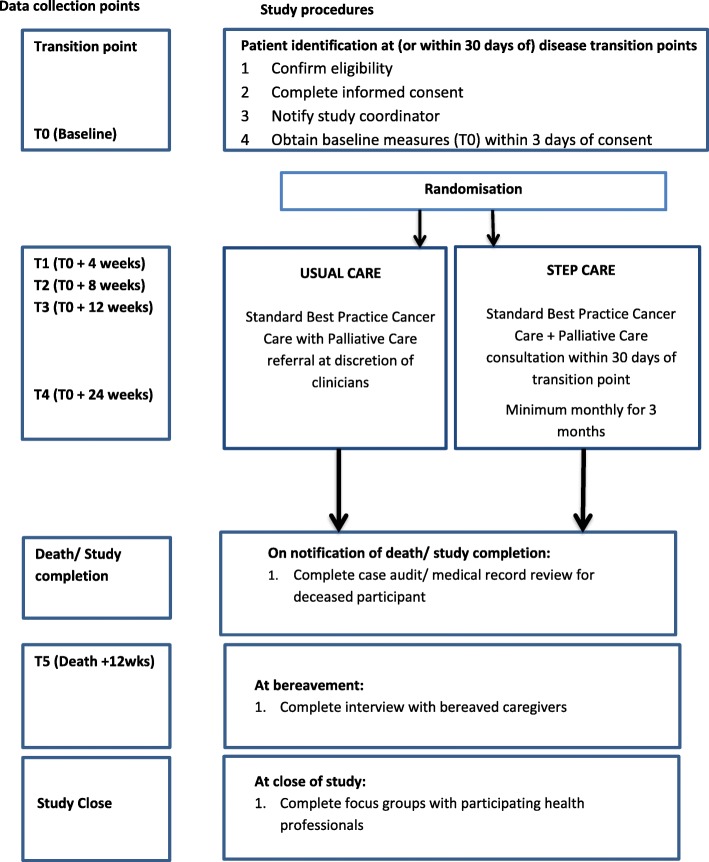
Study schema

Data collection will involve mixed methods including quantitative and qualitative. Study schema and the timing of data collection points are detailed in Fig. 1.

Patients and carers will complete validated self-report measures at enrolment (T0) and monthly thereafter by post until death/study completion (Table 3). Data collection will be coincided with STEP Care visits for those patients randomised to the intervention arm.

**Table 3:** Study outcome measures examining efficacy of intervention

Domain (instrument)	Details of instrument	Time
Patient outcomes		
Quality of life	QUAL-E [40]	Time 0 T1 (+ 4 weeks) T2 (+ 8 weeks) T3 (+ 12 weeks) T4 (+ 24 weeks)
Health-related quality of life and symptom impact	EORTC QLQ-C30 [41, 42]	
Mood	DASS-21 [43]	
Performance status	Australian-modified Karnofsky Performance Status (AKPS) (http://www.bmcpalliatcare.biomedcentral.com/articles/10.1186/1472-684X-4-7)	
Illness understanding	Prompted via nurse-led diary [44]	
Overall survival	Months	T 5 (Following patient death)
Family carer outcomes		
Quality of Life	CQOL-C [45]	Time 0 T1 (+ 4 weeks) T2 (+ 8 weeks) T3 (+ 12 weeks) T4 (+ 24 weeks)
Mood	DASS-21 [43]	
Preparedness to care	PCS [46]	
Satisfaction with care	FAMCARE [47]	T 5 (+ 12 weeks following patient death)
Quality end-of-life indicators in last 30 days of life [47]		
Hospital and ICU days	Patient medical record hospital/ monthly nurse-led diary	Following patient death
Number of emergency-department visits		
Chemotherapy use		
Place of death		
Cost-utility and resource allocation outcomes		
Patient-reported, cost-utility weights, measured by a cancer specific multi-attribute utility index	QLU-C10D [48]	Study completion
Quality-adjusted life years	QALYs	
Incremental cost-effectiveness ratio	Actual cost ($) per unit of change in each outcome/health state/QALY gained	

In addition, demographic, clinical, feasibility and health service data will be collected from patient medical records, Medicare records, nurse-led diaries and qualitative exit interviews with participating clinicians and carers following bereavement (Table 4). Each month, the research nurse will complete a diary involving collection and standardised reporting of assessed performance status, illness understanding and use of health services. Service data prompted for will include hospital stays, use of emergency department, general practitioner visits, community or allied health visits including community palliative care services. Specific details of health service use provided by patients and carers will be confirmed with the administering service where required (Appendix 1).

**Table 4:** Measures of study feasibility

Domain	Measure	Unit of measure
Feasibility		
Number of participants	Identified as eligible	Number
	Consented to participate	Percentage
	Completion of study	Percentage
Step care delivery	Number of initial STEP consultations completed within 14 days planned timeframe	Percentage within 14 days
	Number of interactions per patient	Number consultations
	Time from enrolment to first step interaction	Days
	Completion of outcome measures	Percentage of missing data
	Fidelity of step delivery between recording and documented activities using NAT:PD-C [39, 40]	Percentage content correlation within random audit of 20% of consultations
Acceptability of Step Care		
To patients and carers	Number of withdrawals from STEP Care intervention	Percentage
	Number of adverse events arising from STEP Care intervention	Percentage
	Qualitative interviews with STEP Care family carers following bereavement	Qualitative data
Acceptability of Step Care to clinicians		
To clinicians	NAT: PD-C documentation completion rate by participating STEP care physicians	Percentage of content recorded for consultations conducted
	Focus group with STEP Care physicians regarding perspectives on STEP care content, timing and frequency of interaction	Qualitative data

### Qualitative data collection

Qualitative focus groups will be conducted with health professionals at the study sites to explore the perceived impacts and benefits of STEP Care. In particular, the discussion will explore the views of the timing of STEP Care, the barriers and benefits of its implementation, content, frequency and minimum dose considered meaningful. These focus groups will be recorded, transcribed and analysed for impacts, benefits and additional content.

Interviews with a sample of approximately 15 bereaved family carers will be conducted. The aim of these interviews is to ascertain carers’ reflections associated with the provision of palliative care; specific feedback will be sought including their preferences for the timing of the introduction of palliative care. On their time three questionnaires, a question about willingness for follow-up interview will be listed with a yes/no response format and an open-ended space which request a phone number and best time to call. Of those who express willingness, one in three carers will be approached to invite continued willingness for interview either after the patient’s death or at completion of 3 months of data collection. Interviews following a semi-structured format may be conducted at the hospital or over the telephone and will be recorded and transcribed.

### Planned analyses

Feasibility and health utilisation outcomes will be summarised with descriptive statistics, including frequency counts and percentages (categorical variables) and mean/standard deviation or median/interquartile range (continuous variables) as appropriate.

Preliminary efficacy outcomes for patients (QOL, symptom impact, mood, performance status) and carers (QOL, mood, preparedness, care satisfaction) will be compared between treatment groups, after adjusting for baseline levels using analysis of covariance (ANCOVA). Separate ANCOVA models will be fitted for patients and carers. Results will be presented as estimates and 95% confidence intervals.

From our understanding of the sites, we estimate that the target sample size is *n* = 30 patients/carers per treatment group and *N* = 90 patients/carers overall total. This aligns to an anticipated achievable recruitment rate of approximately two patients/carers per month, per site, over 24 months. Ideally, the aim is for this total of 90 to be distributed equally by tumour type and site based on the minimisation protocol as part of the randomisation process.

A cost-utility analysis will be conducted whereby preference algorithms validated in an Australian population are applied to a cancer-specific, multi-attribute utility index (QLU-C10D) [40] calculated from patient-reported QOL scores (QLQ-C30) [41, 42]. Utility weights will be used to calculate quality-adjusted life years (QALYs). An incremental cost-effectiveness ratio (ICER)—additional costs incurred by intervention divided by any potential effectiveness gained from the intervention—will be calculated to determine cost ($) for each unit of outcome gained. ICERs will be referenced against established UK cost-per-QALY thresholds [43] set by the National Institute for Health and Care Excellence to determine ‘cost-value’ of medical interventions.

Qualitative analysis of interviews and focus groups with health care professionals will be undertaken using a thematic approach consistent with the goal of understanding feasibility, facilitators and barriers in provision of structured integrated palliative care. Interviews with bereaved family carers will be informed by a thematic framework, with flexibility to explore and understand new ideas as they emerge within the data.

## Discussion

As cancer incidence rises, cost-effective models of care that improve quality/equity of care for patients/carers are increasingly critical. Despite treatment breakthroughs, 29 advanced cancer-related deaths occur daily in Victoria, Australia, and 8.2 million deaths annually worldwide (http://www.cancerresearchuk.org/health-professional/cancer-statistics/worldwide-cancer/mortality). Those with advanced disease report high needs and distress that are inadequately addressed. Meanwhile, palliative care which explicitly addresses these concerns [11–17], is accessed variably and late [15, 34, 38]. STEP Care addresses the barriers to engagement with palliative care through routine time of introduction thereby reducing variability and standardisation of delivery of care.

The establishment of feasibility and the qualitative data from this study will inform the conduct of a subsequent study. Meanwhile, the key secondary outcome of quality of life and the effect size will enable robust calculation of the sample size of a phase 3 trial of STEP Care versus usual care alone. Therefore, outcomes of this phase 2 study, if successful, will provide strong support for a full phase 3 trial using defined transition points, measuring quality of life outcomes and will facilitate parallel implementation research. This study will address the as yet unresolved issue about the appropriate timing of early palliative care.

## Appendix

### Study measures

Quality of life at the end of life (patient): (QUAL-E [44]). The QUAL-E is a measure of quality of life specifically targeting those with advanced illness, examining four domains: life completion, impact of symptoms, relationships with health care providers and preparation for end of life. It has demonstrated acceptable validity and reliability, is acceptable to patients and shows consistent performance across different demographic and disease groups.

Health-related quality of life and symptom impact (patient): EORTC QLQ-C30 [41, 42]. The EORTC QLQ-C30 is a widely used instrument to measure quality of life in cancer patients. Consisting of 30 items, this includes 5 functional scales (physical, role, emotional, cognitive and social), 3 symptom scales (fatigue, nausea and vomiting and pain), 6 single items of symptoms/other and a global health status/QOL scale. Designed for self-completion, it has been extensively validated for this population [45].

Quality of life (carer): CQOL-C [46]. Caregiver Quality of Life-Cancer is a 35-item instrument which has been developed specifically for family caregivers of people with cancer. Designed for self-completion, it has been shown to have adequate validity, test-retest reliability and internal consistency in this target population.

Mood (patient and carer): Depression, Anxiety, Stress Scale (DASS-21 [47]). The DASS-21 is a short-form of the DASS instrument and consists of three self-report scales measuring the states of depression, anxiety and stress. The scales have high internal consistency and yield meaningful discriminations in a variety of settings, and acceptable construct validity.

Performance status (patient): AKPS [48]. Australian-modified Karnofsky Performance Status (between 10 and 100) is a single score which measures the patient’s overall performance status or capacity to perform usual activities of daily living.

Illness understanding (patient and carer): [49] Developed by Temel and colleagues to assess the patient’s level of information and understanding of the goals of cancer treatment, patients are asked to nominate if their cancer is curable (or not) and the intention of the cancer treatment.

Preparedness to care (carer): PCS [50]. This 8-point scale provides a measure of the degree to which carers feel able and equipped to take on caring roles.

Satisfaction with care (carer): FAMCARE [51] is a 20-item scale developed to measure family members’ satisfaction with delivery of palliative care.

Patient-reported, cost-utility weights, measured by a cancer specific multi-attribute utility index QLU-C10D [40] is calculated based upon the recordings made by patients on the EROTC QLQ-C30 instrument.

*Feasibility and Acceptability measures:*

A. Patient/carer recruitment and retention rates:
  i. Number of identified eligible patients/carers at included hospital sites;
  ii. Number of consenting patients/carers at included hospital sites;
  iii. Number of participants reaching study completion at included hospital sites.
B. Acceptability of STEP Care intervention to patients/carers:
  i. Number of withdrawals from STEP Care intervention;
  ii. Number of adverse events arising from STEP Care intervention;
  iii. Qualitative interviews with STEP Care family carers following bereavement*.
C. Acceptability of STEP Care intervention to participating physicians:
  i. NAT: PD-C documentation completion rate by participating STEP care physicians;
  ii. Focus group with STEP Care physicians regarding perspectives on STEP care content, timing and frequency of interaction.
D. Refinement of STEP Care intervention procedures ensuring future replication:
  i. Percentage of initial STEP Care interactions occurring within planned 14 day timeframe;
  ii. Median total number of STEP Care interactions per patient/carer.
E. Refinement of outcome measures and data collection methods;
  i. To determine an estimate of effect size for potential patient- and carer- reported outcome measures and variance to inform power calculations for planned phase 3 trial.
  ii. Percentage of missing data on patient- and carer- reported outcome measures, i.e. acceptability of selected measures.
F. STEP Care intervention fidelity;
  i. Timing of intervention delivery: median number of days from enrolment to first STEP Care interaction, and subsequent STEP Care interactions.
  ii. Content of intervention: audit of random sample of 20% of audio-recorded consultations and associated documentation using the NAT: PD-C. [39]
